# Examining the Relationship of Clinical and Laboratory Parameters With Infectiousness to *Phlebotomus perniciosus* and Its Potential Infectivity in Dogs With Overt Clinical Leishmaniasis

**DOI:** 10.3389/fvets.2021.667290

**Published:** 2021-05-04

**Authors:** Manuela Gizzarelli, Antonio Bosco, Valentina Foglia Manzillo, Gioia Bongiorno, Riccardo Bianchi, Daniela Giaquinto, Nour El Houda Ben Fayala, Marie Varloud, Alessia Crippa, Luigi Gradoni, Giuseppe Cringoli, Maria Paola Maurelli, Laura Rinaldi, Gaetano Oliva

**Affiliations:** ^1^Department of Veterinary Medicine and Animal Production, University of Naples Federico II, Naples, Italy; ^2^Unit of Vector-Borne Diseases, Istituto Superiore di Sanità, Rome, Italy; ^3^Ceva Santé Animale, Libourne, France

**Keywords:** canine leishmaniosis, infectiousness, xenodiagnosis, clinical signs, *Phlebotomous perniciosus*

## Abstract

Infected dogs are considered the main domestic animal reservoirs for *Leishmania infantum* parasite. Infectiousness to competent phlebotomine vectors has been associated with many factors, the main being the severity of the disease exhibited by infected dogs. This study examines the relationship between different clinical parameters and the infectiousness to colonized *Phlebotomus perniciosus* sand flies having a blood meal on dogs. Data obtained in the present study come from an untreated group of *Leishmania* sick dogs submitted to xenodiagnosis for the evaluation of a spot on insecticide solution. Seventeen dogs were diagnosed as affected by leishmaniasis through clinical examination, immunofluorescence antibody test (IFAT) serology, and loop-mediated isothermal amplification (LAMP). The disease severity (clinical score) was staged by using a numeric value derived from eight clinical and parasitological parameters. Xenodiagnosis was performed on caged dogs exposed for 1.5 h to sand-fly bites. The following parameters related to sand flies were examined: blood feeding (% of blood engorged females), promastigote detection (% of promastigote-positive sand flies), promastigote burden, and the promastigote stage maturation (potential transmissibility rate). Statistical relationship between the clinical score and entomological parameters was investigated, as well as the possible correlation between each clinical and laboratory parameters and sand fly infection/infectivity. The severity of clinical score may influence the blood feeding by, and the probability of promastigote detection in, sand flies; skin lesions seem to be the main factor that influences the rate of blood feeding. Promastigote burden is related to IFAT titer, skin lesions, and clinical score. All entomological parameters are strongly related among them. This study confirms that both *P. perniciosus* infection and infectivity are influenced by a dog's clinical condition.

## Introduction

Phlebotomine sand flies (Diptera: Psychodidae) are the biological vectors of *Leishmania* parasites (Kinetoplastida: Trypanosomatidae), which are transmitted to vertebrate hosts by the bite of blood-sucking females. Phlebotomines become infected when they ingest amastigote forms from peripheral blood or resident skin macrophages of an infected reservoir host. In the gut of competent vectors, amastigotes transform into flagellate forms (promastigotes) which undergo multiplication, migration to the foregut, and maturation to non-dividing metacyclic promastigotes within 7–10 days, ready to be transmitted into the skin of a new host during a subsequent blood meal (1). Several Phlebotomine species of the *Phlebotomus* (*Larroussius*) subgenus in the Old World, and some species of the *Lutzomyia* genus in the New World, are proven vectors of *Leishmania infantum*, the agent of zoonotic visceral leishmaniasis (ZVL) having infected domestic dogs as the main reservoir host (2). Of them, *Phlebotomus perniciosus* represents one of the most abundant and competent vectors in the western Mediterranean basin, including southern Europe (3) and North Africa (4, 5). Other routes of transmission have been demonstrated among dogs, including sexual, vertical, and blood transfusion-borne infections, whose importance in ZVL epidemiology, however, is still under investigation (6). The role of clinically vs. subclinically infected dogs in maintaining the parasite life cycle in ZVL endemic foci is still debated (7, 8). Dogs' infectiousness to competent vectors has been associated with many factors, the main being the severity of the disease exhibited by infected animals. In fact, canine leishmaniasis (CanL) is a progressing disease characterized by a broad spectrum of signs, ranging from asymptomatic/subclinical infection—frequently characterized by negative *Leishmania* serology and general healthy status—to severe, fatal disease condition. The latter is associated to elevated humoral T-helper 2 unbalanced response, leading to diffuse immune complexes deposition in several organs (9). The severity of the clinical picture is often characterized by a large variety of skin lesions which has been associated with the ability to transmit *L. infantum* back to the sand fly population (10–12). The significance of intact skin in transmission to sand flies is less clear (13); however, the description of the persistence of the parasite at sand flies deposition site is interesting, described 6 months after experimental infection via the vector (14). Infectiousness degree to sand flies was also related to the number of parasites measured in the skin by molecular methods (15) and also demonstrated in dogs with doubtful serology results (16). Despite inherent limitations due to technical difficulties, xenodiagnosis represents the only way for the definitive assessment of the infectiousness to vectors by *Leishmania*-infected hosts, eventually allowing estimates on the potential transmissibility of the parasite. When compared to the huge amount of information concerning CanL infection and disease, data deriving from xenodiagnosis studies appear limited and, recently, often associated to the New World vector *Lutzomyia longipalpis* (16). The aim of this study is to increase the knowledge on the significance of the clinical stage and associated parameters in determining the infectiousness of CanL diseased dogs. In particular, the study examines the relationship of different clinical and laboratory parameters, with the infectiousness to colonized *P. perniciosus* sand flies having a blood meal on diseased dogs.

## Materials and Methods

### Study Design

Data obtained in the present study come from an untreated control group of CanL sick dogs submitted to xenodiagnosis for the anti-feeding and insecticidal efficacy evaluation of a spot-on insecticide solution (17). The study was carried out in a facility located in the Regional Center for Parasitic Diseases (CREMOPAR) of Campania region, South Italy. This area has long been known to be endemic for CanL (18). The study protocol was approved by the Ethics Committee of University of Naples Federico II (n. 2016/0106421). All applicable international, national, and/or institutional guidelines for the care and use of animals were followed (i.e., Good Clinical Practice, VICHGL9, 2000; Directive 2010/63/UE; National Legislative Decree 26/2014); owners signed an informed consent. Seventeen owned dogs of different sex, breed, and age were enrolled following the diagnosis of canine leishmaniasis. Dogs were included if they were ≥6 months, were not clinically pregnant, and have not been treated with anti-*Leishmania* drugs and any adulticide, arthropod growth regulator, or any other pesticide or compound with central nervous system (CNS) activity in the 3 months before admission to the study. Dogs were selected among a larger group, based on their behavior, to allow their quiet exposition to sand flies bites, for 1.5 h in the exposure chambers, without trouble. Any dog suffering from life-threatening condition or severe discomfort was not included in the study. The animals were kept under constant veterinary care during the study period, and they were acclimatized to the study site for at least 5 days before the test.

### Sampling Materials

From each dog, blood (two tubes with EDTA and a tube with serum separator gel), two conjunctival swabs, and fine-needle aspiration of lymph nodes were collected. Each blood sample with EDTA was analyzed: one for a complete blood count analysis (CBC) and the second for the loop-mediated isothermal amplification (LAMP) assay. The blood sample in the serum separator tube was centrifuged at 360 *g* for 15 min and divided into two aliquots, one used for biochemical analysis (chemistry panel, protein electrophoresis) and one stored at −20°C until serological analysis.

### Diagnosis of CanL

CanL was diagnosed based on clinical signs attributable to *Leishmania* infection, serology result (immunofluorescent antibody test, IFAT), and specific LAMP performed on blood, lymph-node, and conjunctival samples. IFAT was performed following the procedures provided by the National Reference Center for Leishmaniosis (CReNaL, Palermo, Italy) to detect anti-*Leishmania* antibodies. The antigen used by CReNaL consisted of promastigotes of the international reference strain for *L. infantum* MHOM/TN/80/IPT1. Serum samples were considered positive if they showed a titer ≥1:160; positive and negative controls provided by CReNaL were added for each test to verify the validity of the results. DNA extractions from blood and lymph nodes were performed using the Leishmania Screen Glow kit (Avantech Group, Angri, SA, Italy) following the manufacturer's instructions. For conjunctival swabs, DNA extraction was performed from one of the two samples using the above kit and procedure with slight modifications. Briefly, the conjunctival swab was put in a 2 ml tube with 500 μl of extraction buffer for 10 min at room temperature. An aliquot of each extracted DNA sample was stored at −20°C until LAMP analysis. LAMP was performed using the above-mentioned kit, following the manufacturer's instructions. In each amplification run, one positive and one negative control (without DNA), both supplied by the kit, was used. Specific primer were used to amplify the 18S small subunit rRNA gene, plus a partial sequence of the internal transcribed spacer 1 (ITS-1) (19).

### Disease Severity Score

To be considered eligible to the study, dogs had to be positive by IFAT *Leishmania* serology and by LAMP-PCR performed on at least one of three different matrices. CanL severity was staged using a numeric value (range: 0–16) derived from eight clinical and parasitological parameters, including clinical signs, clinicopathological alterations, IFAT value, and LAMP result (Table 1). The severity of skin pathology was defined by the “skin score” (range: 0–8), obtained by adding scores attributed to each cutaneous sign detected on dogs.

**Table 1:** Clinical and parasitological parameters to assess the disease severity score.

**Parameter**	**Score**
**IFAT value**	
≤ 1:320	1
>1:320	2
**LAMP-PCR**	
1 positive tissue	1
>1 positive tissue	2
**Clinical signs (not cutaneous)**	
Absence	0
Presence	1
**Cutaneous signs**	
Absence	0
Focal/multifocal alopecia	1
Diffuse alopecia	2
Dermatitis /nodules	2
Ulcers	3
**Hematological disorders**	
Absence	0
Presence	1
**Elevated renal parameters**	
Absence	0
Presence	1
**Elevated liver enzymes**	
Absence	0
Presence	1
**Abnormal protidogram**	
Absence	0
Presence	1
Disease severity	(0–16)

### Sand Flies and Xenodiagnosis

Adult *P. perniciosus* specimens were from a Spanish strain maintained under laboratory conditions since June 2012 at the facilities of Istituto Superiore di Sanità, Italy, and certified for pathogen-free status. Standard rearing conditions were 28 ± 1°C and 75–80% relative humidity (RH), with an inverted photoperiod of 17 h light/7 h dark to facilitate the feeding behavior of females during daytime. Dogs were exposed for 90 min to the bites of 3- to 9-day-old unfed *P. perniciosus* females (a range of 85–141 in different experiments) with the addition of about 10% males to promote biting behavior. The exposure cages have been designed to accommodate individual dogs of different sizes (90 cm high, 100 cm long, 75 cm wide). The bottom and the lower side of the cage's walls were made of polymethyl methacrylate (Perspex®) to avoid breakages by the dogs, whereas the upper side of the walls and the roof were made of fine-gauze cloth. Two fine-gauze tunnels were fixed to the cage to allow dog handling and sand fly release/recovery, respectively. Two hours before the sand fly exposure, Adaptil® Express Calming tablets (CEVA, FRANCE) were administered to help dogs to relax during the test. These tablets solely contain dog-appeasing pheromones (DAPs) which are not considered pharmacological compounds. This product was used instead of sedative drugs to avoid a decrease in the dogs' temperature, which would negatively affect sand-fly biting behavior. The dogs were introduced into the cage 30 min before release of the sand flies to acclimatize them. During acclimatization and for the entire duration of exposure, the caged animals were maintained in the dark. Standard conditions suitable for sand flies were reproduced in the test room with temperature in the range of 25–28°C and RH close to 50%. At the end of the test the light was turned on, the dog brought out of the cage, and the sand flies, both alive and dead, were recovered with the aid of a mouth aspirator. The specimens were pooled in cylindrical plastic pots (400 ml) fitted with a tight lid, which was perforated, with the hole covered with a fine gauze holding a piece of cotton soaked with glucose-saturated solution, and maintained thereafter at the usual rearing conditions. Blood feeding rate (no. of engorged specimens/no. recovered) was assessed at 24 h post exposure, and live engorged sand flies were then individually transferred into 5-ml glass vials. Promastigote detection, in order to calculate sand fly infection rate and hence dog's infectiousness, and assessment of promastigote burden and stage maturation in positive flies, in order to estimate a potential transmissibility rate, were performed through dissection and microscopic examination of gut samples from live blood-fed sand flies at 96 h post blood meal. This period is necessary for the initial, successful parasite multiplication and foregut migration in the invertebrate host, which are the prerequisites for maturation and subsequent transmission of metacyclic stages (1). The burden of *Leishmania* infection was evaluated as light (<100 parasites/gut = score 1), moderate (100–500 parasites/gut = score 2), heavy (500–1,000 parasites/gut = score 3) or very heavy (>1,000 parasites/gut = score 4) (20).

### Statistical Analysis

The correlation analysis between the dichotomous variables of laboratory score (IFAT, LAMP-PCR, hematological disorders, elevated liver enzymes, elevated creatinine, and abnormal protidogram) and sand fly infection (feeding rate, promastigote detection, and promastigote burden) was evaluated using the Kruskal–Wallis test. The correlation of ordinal variables of clinical/laboratory score (clinical signs except cutaneous ones, cutaneous signs, and disease severity) and quantitative morphometric variables (age, weight, and body surface area) with sand fly infection was assessed by Spearman's rho correlation coefficient. In order to understand how all clinical/laboratory score associate with sand fly infectivity (potential transmissibility rate), the Pearson's chi-squared test for dichotomous and ordinal variables and the Spearman's rho correlation coefficient (*r*_*s*_) for quantitative variables were performed. In addition, the correlation between entomological parameters (feeding rate, promastigote detection, promastigote burden, and potential transmissibility rate) by Spearman's rho correlation coefficient was investigated. All statistical analyses were performed using SPSS Statistics v.23 (IBM, Armonk, NY, USA), and significance level of *p* < 0.05 was applied.

## Results

The disease severity score of the 17 enrolled dogs, summarized in Table 2, ranged between 5 and 14 (median: 10). All dogs exhibited clinical signs related to CanL, such as lymph node enlargement, cutaneous lesions, weight loss, muscular hypotrophy, or ocular involvement. Skin lesions were present in 81% of the cases, classified as focal/multifocal alopecia (35.2%), diffuse alopecia (47.0%), furfuraceous dermatitis (41.2%), and ulcers (29.4%). Hematological disorders, mainly represented by non-regenerative anemia, together with hyperglobulinemia and hypoalbuminemia, were the most frequent clinicopathological findings. The severity of the clinical picture was always associated to elevated IFAT titers that ranged 1:160–1:5,120. The main entomological findings related to each single dog are reported in Table 3. All dogs demonstrated adequate attractiveness to sand flies, as shown by the median value of 55.4% blood feeding rate (range: 14.3–90.7%). Seven dogs were not infectious to sand flies; these dogs were characterized by lower median blood feeding rate, when compared with the remaining 10 dogs (35.6 vs. 69.7%; *r*_*s*_: 0.574, *p* < 0.05). Infectious dogs determined an infection rate ranging from 17.5% (Dog 4) up to 78.9% (Dog 9) of sand flies that had a blood meal on them. Based on the assessment of promastigote development in sand fly gut at 96 h post blood meal, the potential transmissibility rate of *L. infantum* by the infectious dogs ranged from 7.5% (Dog 4) to 63.6% (Dog 6). Statistical analyses of the relationship of canine clinical and laboratory parameters with sand fly blood feeding and sand fly infection parameters are reported in Table 4. These findings suggest that severity of clinical score is not only the main determinant for infectiousness of dogs toward the vector, but also for potential infectivity of sand flies that had a blood meal on them. The presence of skin lesions seems to be the main parameter that influences dog's infectiousness. Interestingly, sand fly infectivity is significantly related to IFAT titer. It is worthy to note that all entomological parameters are strongly related among them (*p* < 0.05; *r*_*s*_ *range* from 0.574 to 0.971), but apparently none of them is associated with morphometric variables of dogs (*p* > 0.05).

**Table 2:** Breed, age, sex, and disease severity of the enrolled dogs.

**Dog (n.)**	**Identification**			**Score**					
	**Breed**	**Age (ys)**	**Sex**	**IFAT**	**PCR**	**Cutaneous signs**	**Clinical signs**	**Laboratory abnormalities**	**Disease severity**
1	Mongrel	3	M	2	2	3	1	2	10
2	En. Setter	8	M	2	1	7	1	2	13
3	Mongrel	6	M	2	2	4	1	3	12
4	Mongrel	6	M	2	1	7	1	2	13
5	Mongrel	7	F	2	2	7	1	2	14
6	Ep. Breton	4	M	2	2	1	1	2	8
7	Mongrel	3	M	2	1	4	1	1	9
8	Mongrel	3	F	2	2	6	1	2	13
9	Mongrel	1	M	2	1	4	1	2	10
10	Mongrel	4	M	1	0	4	1	1	7
11	Mongrel	9	F	2	1	1	1	2	7
12	Mongrel	2	M	2	1	1	1	1	6
13	Mongrel	6	M	2	1	4	1	2	10
14	Mongrel	5	M	1	2	0	1	2	6
15	En. Setter	5	F	1	1	0	1	2	5
16	Mongrel	8	F	2	2	5	1	2	12
17	Mongrel	4	F	2	1	0	1	1	5

**Table 3:** Xenodiagnosis results: sand flies feeding rate and promastigote infection.

**Dog no**.	**Feeding rate (%)**	**Promastigote detection (%)**	Promastigote burden (score)^*^	**Potential transmissibility rate (%)**
1	90.7	58.3	3	52.8
2	84.9	69.1	3	59.6
3	84.4	25.5	2	19.6
4	82.3	17.5	2	7.5
5	78.0	70.0	3	60.0
6	75.7	72.7	2	63.6
7	72.2	0	0	0
8	63.6	39.3	2	30.3
9	61.9	78.9	2	50.0
10	54.2	0	0	0
11	44.8	0	0	0
12	39.3	50.0	2	36.4
13	36.5	40.7	2	27.8
14	24.4	0	0	0
15	21.1	0	0	0
16	14.4	0	0	0
17	14.3	0	0	0

* *Promastigote burden was calculated as the parasitic score average for each sand fly*.

**Table 4A and B:** Analysis of the relationship of laboratory and clinical dog's parameters with sand flies' parameters.

**(A)**			
**Dog variable**	**Sand fly variable**		
Laboratory parameters^*^	**Feeding rate**	**Promastigote detection**	**Promastigote burden**
IFAT	*p* = 0.308	*p* = 0.139	*p* = 0.139
Hematological disorders	*p* = 0.308	*p* = 0.543	*p* = 0.456
Elevated liver enzymes	*p* = 0.654	*p* = 0.765	*p* = 0.323
Elevated creatinine	*p* = 0.541	*p* = 0.478	*p* = 0.487
Abnormal protidogram	*p* = 0.534	*p* = 0.898	*p* = 0.952
Clinical/lab parameters^**^			
Absence of cutaneous signs	ND	ND	ND
Cutaneous signs	***p*** **=** **0.024**	*p* = 0.179	*p* = 0.152
Disease severity	***p*** **=** **0.009**	*p* = 0.058	***p*** **=** **0.044**
PCR	*p* = 0.363	*p* = 0.323	*p* = 0.172
Morphometric parameters^**^			
Age	*p* = 0.981	*p* = 0.368	*p* = 0.539
	*r_*s*_* = −0.006	*r_*s*_* = −0.233	*r_*s*_*= −0.160
Weight	*p* = 0.670	*p* = 0.485	*p* = 0.641
	*r_*s*_* = −0.112	*r_*s*_* = −0.182	*r_*s*_*= −0.122
Body surface area	*p* = 0.687	*p* = 0.466	*p* = 0.637
	*r_*s*_* = −0.106	*r_*s*_* = −0.190	*r_*s*_* = −0.123
**(B)**			
**Dog variable** Laboratory parameters^*^	**Sand fly variable** ** Transmissibility potential**		
IFAT	***p*** **=** **0.043**		
	**Cramér's** ***V*** **=** **0.553**		
Hematological disorders	*p* = 0.284		
	Cramér's *V* = 0.274		
Elevated liver enzymes	*p* = 0.165		
	Cramér's *V* = 0.540		
Elevated creatinine	*p* = 0.585		
	Cramér's *V* = 0.299		
Abnormal protidogram	*p* = 0.856		
	Cramér's *V* = 0.369		
Clinical/lab parameters^*^			
Absence of cutaneous signs	ND		
Cutaneous signs	***p*** **=** **0.032**		
	**Cramér's** ***V*** **=** **0.758**		
Disease severity	***p*** **=** **0.014**		
	**Cramér's** ***V*** **=** **0.761**		
PCR	*p* = 0.190		
	Cramér's *V* = 0.268		
Morphometric parameters^**^			
Age	*p* = 0.433		
	*r_*s*_* = 0.245		
Weight	*p* = 0.383		
	*r_*s*_* = 0.235		
Body surface area	*p* = 0.365		
	*r_*s*_* = 0.236		

* Kruskal-Wallis test in **(A)**, ^*^Chi-square test in **(B)**.

** *Spearman's rho correlation coefficient*.

*ND, not detected*.

*Bold values represent the significant values*.

## Discussion

Xenodiagnosis is established as the best method to determine the role of domestic and wild animals, and humans, to act as reservoir hosts for *Leishmania* species (21). Therefore, the accurate evaluation of dog's infectiousness remains problematic due to such unpractical approach, which is only possible by specialized institutions. Specific serology and PCR tests that detect *L. infantum* infection in dogs seem to have low usefulness in providing information on the capacity of infected dogs to be infectious (22), even if some studies positively correlate the dog's skin and blood parasitic load with the capacity to infect sand flies vectors (23). Several limiting factors, first of all the need for mass breeding of sand fly colonies, make xenodiagnosis not easily applicable to large-scale studies, in particular to evaluate dog's infectiousness during and after the use of preventative and/or therapeutic compounds against *Leishmania* parasites (24–27). Because of the complex methodology, different ways to perform xenodiagnosis could make it difficult to compare results from different investigations, which would suggest the need for a consensual standardization. For example, the main differences across published studies are the number of sand flies employed for dog biting, the nature and extension of the body surface exposed to their bite (for example, the whole body or the head or areas limited to the ear, neck, or belly), and the time of exposure. In the present study, we tried to mimic a natural condition of sleeping dogs, for example, by avoiding deep anesthesia or sedation that could influence physiological parameters such as body temperature, and by giving to female sand flies the opportunity to bite on the whole body surface. The use of natural pheromones to appease our dogs during xenodiagnosis gave very good results. All enrolled dogs were bitten by sand flies with no trouble during their caged period. They appeared in a sleeping-like condition, similar to what happens during the natural nocturnal activity of phlebotomine sand flies. Our study included only CanL sick dogs as per trial design, so that we could not investigate on the infectiousness and related entomological parameters of the existing broad spectrum of canine infections. In fact, further limitations in the definition of dog's infectiousness are due to a non-definitive proof of the role of subclinically infected dogs (i.e., asymptomatic) in comparison with clinically ill animals in the contribution of ZVL transmission risk. Some studies pointed out the role of asymptomatic dogs and/or the lack of correlation with clinical signs for determining the dog's infectiousness (11, 23, 28–31). However, the majority of studies seem to confirm that sick dogs are more dangerous epidemiologically and that the severity of clinical signs may directly influence the sand fly infection rate (32–36). Contradictory interpretation of results from different studies is likely to be due to the lack of standardized definition of the subclinical/asymptomatic condition, as often it is only referred to as the absence of overt clinical signs at external inspection without considering the possible presence of clinicopathological alterations. Analogously, there is no definitive consensus for the definition of clinical stages in diseased dogs, although in the past years the Leishvet and Canine Leishmaniotic Working Group (CLWG) groups classifications 112, (37, 38) have been considered the most applicable in the clinical practice (39). In our study, we chose a different clinical score for statistical purposes. Our results clearly confirmed that the dog's infectiousness to *P. perniciosus* is significantly determined by disease severity, with an elevated antibody level measured through IFAT as the most important marker related to infectiousness toward *P. perniciosus* and associated with high probability of parasite transmissibility. This last finding confirms that the spread of parasite during *Leishmania* infection is strongly associated to the negative role of an unbalanced immunological Th2-humoral response, causing immune-complexes deposition and tissue damage in different body compartments (9). Dogs' morphometric parameters such as age, body surface, and weight did not influence the reservoir potential of the infected animals, similarly to what resulted for each single clinical parameter. Once again, these results demonstrate how difficult it is to predict the infectiousness of infected dogs based on the host morphological characteristics or one or a few clinical and/or laboratory parameters. Undoubtedly, the skin represents a remarkable parasite's rich tissue during progressive *Leishmania* infection, which explains why parasites can also be isolated from intact skin of naturally infected dogs (13). In addition, an experimental model carried out on beagle dogs exposed to infected *L. longipalpis* using custom-made feeders applied to the neck or belly revealed the persistence of the parasite in small-positive lesions or intact skin distal from them for many months (14). Skin lesions observed in clinically ill dogs have been associated with the ability to transmit *L. infantum* back to the sand fly population (10–12). Among these dogs, those with dermatitis resulted much more infectious in a study evaluating serological test as predictive marker for infectiousness (22). In our study, skin lesions represented the most frequent clinical sign observed in the enrolled dogs. The kind and severity of dermatological alterations are indicative of the chronicity of the disease, as described in a previous paper (40). Many animals exhibited the simultaneous presence of more than one skin lesions, usually due to mechanical trauma and/or vascular changes (41, 42). The most common histologic lesion is referred to as a pyogranulomatous inflammation that affects different structures of the skin and, less often, by immune complex deposition (43). The presence of parasites in the skin is originated by hematogenous dissemination, whereas nodules and papules are regarded as representing sites of *Leishmania* promastigote deposition (44). It is not surprising that in our study a marked association between skin lesions and dog's infectiousness was demonstrated. Unfortunately, we did not have owners' consent for skin biopsy that could have added more information on individual parasite loads associated with different lesions. Even though all dogs were bitten by sand flies, 7/17 were not infectious to the vector, independently from their individual clinical status. It is worthy to note that feeding rates as high as 45–72% were recorded for three of these dogs, which means that there were good chances for a parasite to be picked up from any approachable part of the body. The reasons for which it happens in natural disease conditions remain unclear. Our experimental conditions of xenodiagnosis did not permit monitoring of full parasite development in the vector for 7–10 days—i.e., till metacyclogenesis recording—because of elevated insect mortality after 4–5 days from the blood meal. Anyhow, *P. perniciosus* infectivity expressed as promastigote multiplication and foregut migration at 96 h from biting (potential transmissibility) was clearly related to the same clinical and parasitological parameters associated to dog's infectiousness; moreover, all examined entomological parameters were statistically related among them. This study confirms that both *P. perniciosus* infection and infectivity can be influenced by a dog's clinical condition, particularly the skin lesions. Veterinarians, public health authorities, and dog owners should be aware of this risk for recommending and applying the necessary measures to prevent *Leishmania* spreading, primarily consisting in the application of sand fly repellent pyrethroid formulations which, unfortunately, are rarely used in sick dogs. The use of anti-*Leishmania* therapeutic protocols is known to reduce the parasite load and hence infectiousness by treated dogs but have only temporary efficacy.

## Data Availability Statement

The raw data supporting the conclusions of this article will be made available by the authors, without undue reservation.

## Ethics Statement

The animal study was reviewed and approved by Ethics Committee of University of Naples Federico II. Written informed consent was obtained from the owners for the participation of their animals in this study.

## Author Contributions

MG, LG, GC, LR, MV, and GO contributed to the conception and design of the study. NB, AC, and DG organized the database. GO, LG, VF, and GB wrote the manuscript. LG, GB, and RB performed the entomological part of the study. MG, DG, VF, AB, and NB performed the clinical examination and clinical laboratory analyses. MM and AB performed the statistical analysis. All authors contributed to manuscript revision and read and approved the submitted version.

## Conflict of Interest

MV and AC were empolyed by Cevà Santé Animale. The remaining authors declare that the research was conducted in the absence of any commercial or financial relationship that could be construed as a potential conflict of interest.
